# Biomechanical investigation of the type and configuration of screws used in high tibial osteotomy with titanium locking plate and screw fixation

**DOI:** 10.1186/s13018-019-1062-8

**Published:** 2019-01-28

**Authors:** Yen-Nien Chen, Chih-Wei Chang, Chun-Ting Li, Chih-Hsien Chen, Chi-Rung Chung, Chih-Han Chang, Yao-Te Peng

**Affiliations:** 1 Department of BioMedical Engineering, National Cheng Kung University, No.1, University Road, Tainan City, 701 Taiwan; 2 Department of Orthopedics, Show Chwan Memorial Hospital, Changhua City, Taiwan; 3 Department of Orthopedics, College of Medicine, National Cheng Kung University, Tainan City, Taiwan; 4 Department of Orthopedics & Joint Reconstruction Center, National Cheng Kung University Hospital, Collage of Medicine, National Cheng Kung University, Tainan City, Taiwan; 5 Graduate Institute of Mechatronic System Engineering, National University of Tainan, Tainan City, Taiwan; 6 Department of Orthopaedic Surgery, Tainan Municipal Hospital (Managed by Show Chwan Medical Care Corporation), Tainan City, Taiwan; 7 Department of Orthopedics, Chi-Mei Medical Center, Tainan City, Taiwan; 8 Metal Industries Research & Development Centre, Kaohsiung City, Taiwan

**Keywords:** High tibial osteotomy, Open wedge, Screw stress, Far-cortical locking screw, Finite element method

## Abstract

**Background:**

To maintain the corrected alignment after high tibial osteotomy (HTO), fixation with titanium locking plate and screws is widely used in current practice; however, screw breakage is a common complication. Thus, this study was to investigate the mechanical stability of HTO with locking plate and various screw fixations, including the length as well as the type.

**Methods:**

A finite element (FE) model involving a distal femur, meniscus, and a proximal tibia with HTO fixed with a titanium locking plate and screws was created. The angle of the medial open wedge was 12°, and bone graft was not used. Two types of screws, namely conventional locking and far-cortical locking screws, with various lengths and configurations were used. At the proximal tibia, conventional locking screws with different lengths, 30 and 55 mm, were used; at the tibia shaft, different screw fixations including one-cortical, two-cortical, and far-cortical locking screws were used.

**Results:**

The use of far-cortical locking screw generated the highest equivalent stress on the screws, which was four times (from 137.3 to 541 MPa) higher than that of the one-cortical screw. Also, it led to the maximum deformation of the tibia and a greater gap deformation at the osteotomy site, which was twice (from 0.222 to 0.442 mm) larger than that of the one-cortical screw. The effect of different locking screw length on tibia deformation and implant stress was minor.

**Conclusion:**

Thus, far-cortical locking screws and plates increase interfragmentary movement but the screw stress is relatively high. Increasing the protection time (partial weight duration) is suggested to decrease the risk of screw breakage in HTO through fixation with titanium far-cortical locking screws and plates.

## Introduction

High tibial osteotomy (HTO) with medial opening wedge is a surgical technique to reconstruct the bony structure of knees and correct their mechanical axis [1, 2]. This technique may be used to manage the early stage of knee osteoarthritis (KOA), particularly in relatively young patients (40–60 years) [3, 4]. Currently, HTO with locking plate and screws is popular because it efficiently slows the progressive degeneration of the knee joint and holds the requirement of terminable joint replacement [5, 6].

To maintain the corrected alignment after HTO, the use of locking plate and screws helps to establish a strong support for the osteotomied tibia till bone union [7–9]. In current practice of HTO, traditional titanium locking plate and screw fixation yields acceptable outcomes, and approximately 90% of the patients who returned to work or sport did so within 1 year [6]. However, some complications following HTO such as screw breakage or loss of reduction are not uncommon [10]. To obtain better results, further studies focused either on the structural stability established between the bone and plate or on the creation of beneficial biomechanical stimuli from interfragmentary movement via flexible/dynamic fixation were made in the past [11–13]. In these biomechanical studies, some issues including the geometry and configuration of plate, the use of wedge spacer, and even the interfragmentary movement promoting callus formation during bony healing [7, 11, 14–16] were investigated. However, to our note, a more fundamental factor, the screw, was less mentioned and rarely investigated. In addition, considering the major clinical issue about screw breakage, it is worth further analyzing and demonstrating the screw stress in HTO-related studies.

Nevertheless, it is difficult to explore the internal stress of screws, plates, and bones, because no sensor is sufficiently small to insert into the body without disturbing the responses. By contrast, the finite element (FE) method, a numerical simulation based on the loading condition and measured material properties of the bone and metallic implants, helps to calculate the internal stresses and has been used in many biomechanical studies [17–20]. Therefore, this study aimed to explore the mechanical stability and screw stress of HTO fixed with titanium locking plate and screws comparing various lengths and types (flexible or traditional) of locking screws using the FE method.

## Methods

### Solid model

An intact solid knee joint model consisting of the tibia, distal femur, and meniscus was directly created using open-source computed tomography images of the Visible Human Project (from National Institutes of Health, https://www.nih.gov/) in this study. The contours of the bones in each section were retrieved by examining relatively higher gray values than the surrounding tissues using the Avizo Version 6 (VSG SAS, Bordeaux, France) software. Furthermore, the cortical and cancellous bones were demarcated according to their gray values. The three-dimensional bone models were created based on the retrieved contours of each section. The bone models were then imported into the CAD software SolidWorks 2014 (Dassault Systèmes SolidWorks Corp., Waltham, MA, USA) for creating the meniscus cartilage. The space between the distal femur condyles and the proximal tibia was modeled as the meniscus cartilage, and the shape of the meniscus was modified on the basis of a previous study [21]. Then, the HTO model with medial open wedge and metallic locking plate and screw fixation was created in SolidWorks, too. Virtual planes were defined to divide the tibia into two parts, namely the medial wedge and rest of the tibia. The angle of the medial open wedge was set to 12°, and the length of the lateral hinge (apex of the wedge to tibial border) was set to 10 mm [22]. The volume of the medial wedge was then removed in order to mimic the open wedge of HTO without bone graft.

**Fig. 1:**
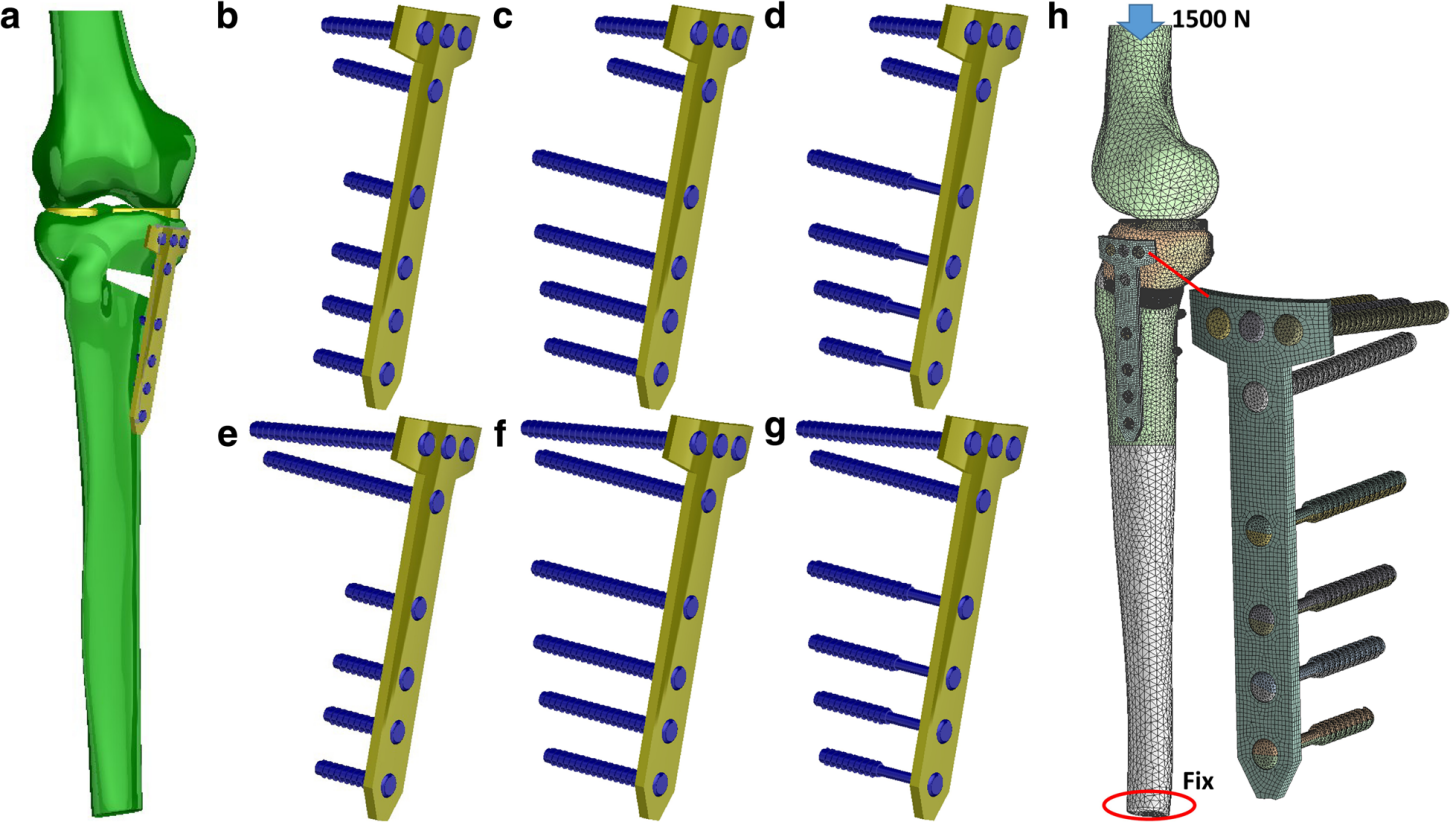
**a** Solid model. Short proximal screw with **b** one-cortical, **c** two-cortical, and **d** far-cortical locking screws. Long proximal screw with **e** one-cortical, **f** two-cortical, and **g** far-cortical locking screws. **h** Boundary condition used in this study

The TomoFix plate (TomoFix, DePuy Synthes, Oberdorf, Switzerland) and locking screws were used to fix the tibia with HTO (Fig. 1a). The length and width of the TomoFix plate were 115 and 16 mm, respectively. The plate thickness was 3 mm. Two types of locking screws were used in this study, namely traditional locking screw and far-cortical locking (flexible fixation) screws. The tibia shaft was fixed using far-cortical locking and traditional locking screws, and the proximal tibia was fixed using a traditional locking screw only. Six types of screw configuration for HTO fixation were analyzed (Fig. 1b–g). Conventional 30- and 55-mm locking screws were used at the proximal tibia (above the wedge), and three types of screw, namely one-cortical (30 mm), two-cortical (35–50 mm), and far-cortical locking screws (35–50 mm), were used for the tibia shaft (below the wedge). The diameter of the traditional locking screws was 5 mm. For the far-cortical locking screw, the outer diameter of the thread part (distal) was 5 mm, and the diameter of the proximal shaft was 3.5 mm [23]. The bone around the proximal screw shaft of the far-cortical locking screw within 0.75 mm was also removed. Then, a 0.75-mm gap between the proximal screw shaft (of the far-cortical locking screw) and the tibia was created, and the screw shaft did not touch the bone during the initial unloading state. The screw insertion into the tibia was mimicked using Boolean operation to ensure that no space existed between the bone and the thread of screws. The thread of the screw head and the screw hole of the plate were simplified as a cylinder.

### FE model

The solid model was then imported into ANSYS Workbench V 17 (Swanson Analysis Systems, Inc., Houston, PA, USA) for generating the mesh and simulation. The ligaments around the knee joint, including medial collateral, lateral collateral, anterior cruciate, and posterior cruciate ligaments, were modeled using tension-only springs (Table 1). These springs were created by linking the origin and inserting sites of the corresponding ligaments. The longitudinal stiffness of these springs was set according to the cross-sectional area, the Young’s modulus obtained from a previous study [24], and the length in the present model. The elastic moduli of the cortex and trabecular bone was set to 12 GPa and 430 MPa, respectively [25, 26], and their Poisson’s ratios were set to 0.3. The Young’s modulus and Poisson’s ratio of the meniscus were set to 100 MPa and 0.1, respectively [25]. The metallic implants, including the plate and screws, were made from titanium, and the plasticity of titanium was utilized. Hence, the Young’s modulus and Poisson’s ratio of titanium in the linear elastic phase were defined to 110 GPa and 0.3, respectively. The yield strength and tangent modulus in the plastic phase were set to 800 and 1250 MPa, respectively. The material properties of titanium were defined according to the engineering database in ANSYS Workbench.

**Table 1:** Material properties of the springs used in this study to simulate ligaments

Ligament	Numbers of spring	Stiffness (N/mm)	Cross section area (mm^2^)	Elastic modulus (MPa)
Medial collateral ligament	2	10.6	1.54	345
Lateral collateral ligament	2	10.6	1.54	345
Anterior cruciate ligament	1	17.5	1.29	345
Posterior cruciate ligament	1	20.6	1.92	345

The thread part of the screws and the surrounding bone were assigned bonding contact behavior, as were the screw head and screw hole of the plate. The contact between the proximal screw shaft of the far-cortical screw and the bone nearby (gap distance of 0.75 mm) was a frictional surface-to-surface contact (if the contact occurred). Furthermore, the contact behaviors between the bones at the osteotomy site were frictional. The contact behavior between the meniscus and the distal femur condyles was frictionless. The friction coefficients of metal-to-bone and bone-to-bone were 0.3 and 0.45, respectively [27]. A 1500 N (about two times the body weight) compressive force was applied on the superior surface of the distal femur to simulate the physiological load of the knee joint in normal gait [22, 28–30]. The degree of freedom of the distal femur was set to zero along the *X* and *Y* axes, and movement was allowed only in the vertical direction (*Z* axis). The distal end of the tibia was fixed (Fig. 1h).

### Validation

**Fig. 2:**
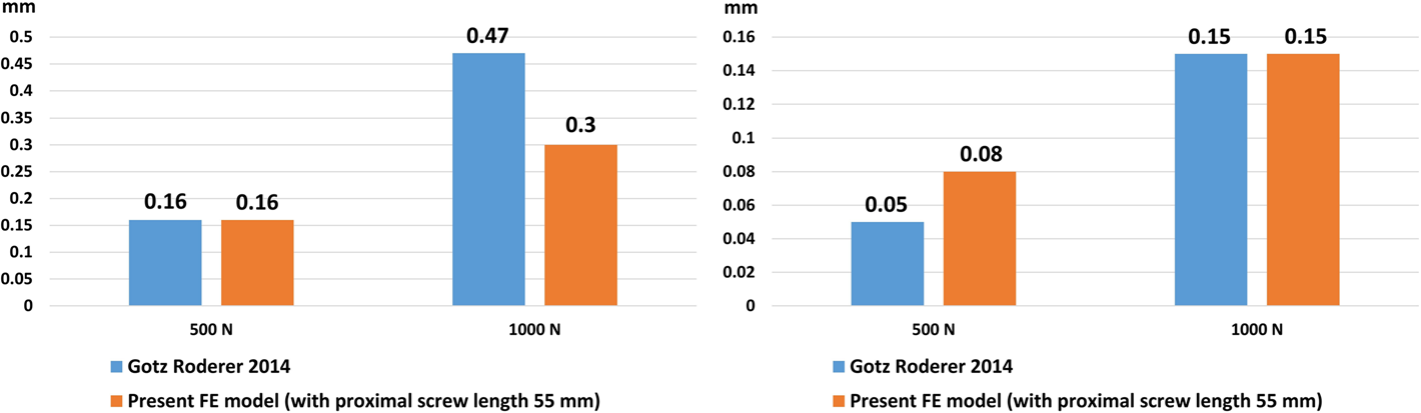
Comparison of interfragmentary movements (mm) with flexible fixation screw (left) and traditional locking screw (right) in the FE model and Roderer et al.’s study

For validation of this FE model, the maximum interfragmentary movements of the FE model with one-cortical and far-cortical locking screws at the tibia shaft (the screw length at the proximal tibia was 55 mm) were compared with those of Roderer et al.’s study [11]. The interfragmentary movement in the FE model and Roderer et al.’s study was similar (Fig. 2). The differences of interfragmentary movement between the present FE model and Roderer et al.’s study were just 0.17 mm (under 1000 N) and 0.03 mm (under 500 N) with the flexible fixation screw and traditional locking screw, respectively.

### Incidence

Equivalent stress (also formulated in terms of the von Mises stress) was used as an index for evaluating stress on the metallic screw and plate, whereas maximum principle stress was used for evaluating stress on the bones. The maximum displacement of the tibia and the deformation of the osteotomy gap were used for analyzing HTO stability.

## Results

**Fig. 3:**
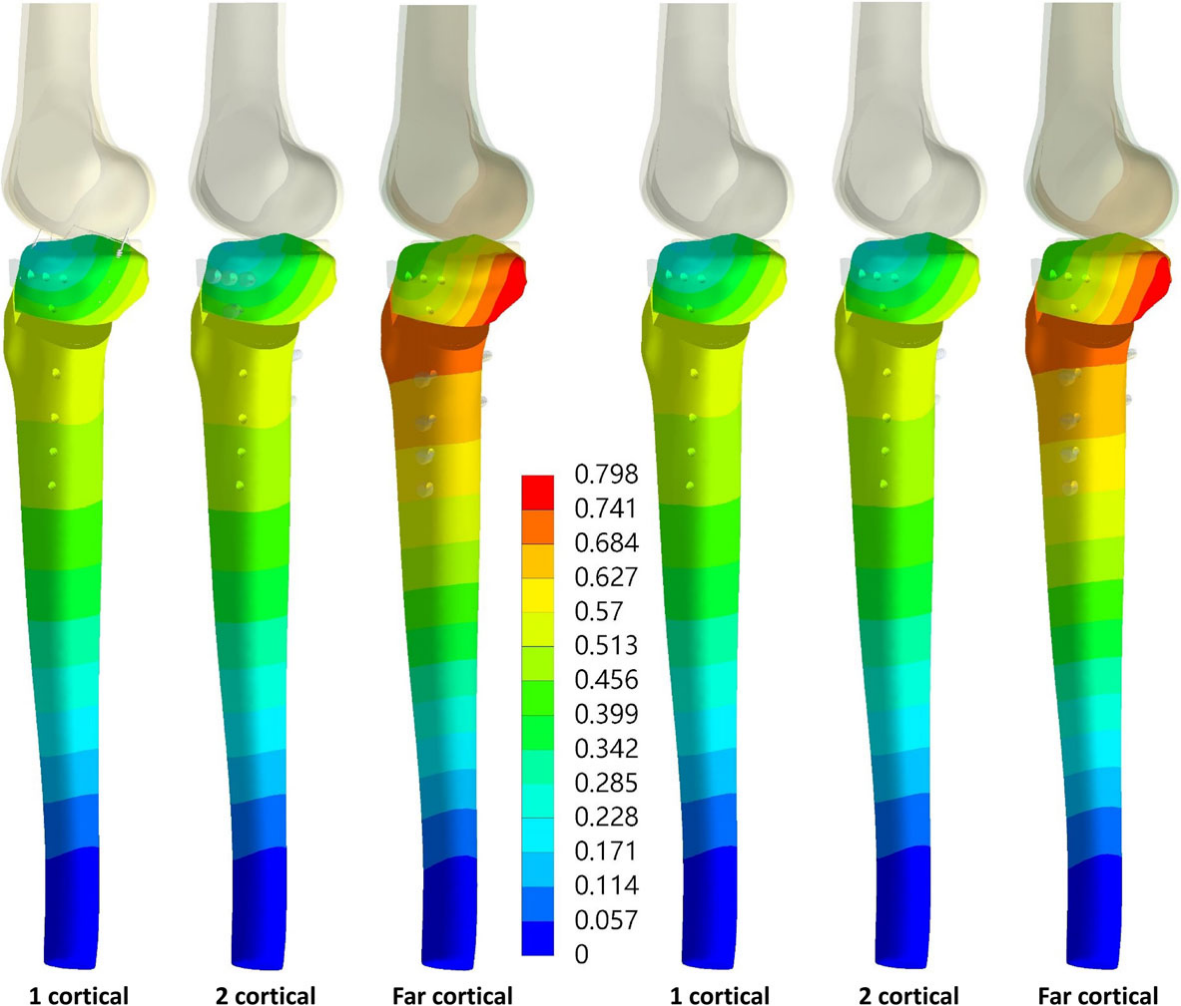
Total displacement of the tibia with short (left) and long (right) screws at the proximal tibia

With identical proximal screw length, the different fixation methods using conventional locking screws on the tibial shaft, one-cortical and two-cortical, shared similar gap deformation as well as the tibia displacement (Table 2). Compared with the conventional locking methods, the use of far-cortical locking screws resulted in a larger tibia displacement as well as gap deformation at the osteotomy site (Table 2 and Fig. 3). The maximum gap deformation with the far-cortical locking screw was 2.03 (from 0.219 to 0.445) and 2.05 (from 0.216 to 0.442) times that of the two-cortical screws when the proximal screw lengths were 30 and 55 mm, respectively.

**Table 2:** Maximum displacement of the tibia, gap deformation, and stress of the implants and bone

	Top screws with length 30 mm			Top screws with length 55 mm		
	One-cortical	Two-cortical	Far-cortical	One-cortical	Two-cortical	Far-cortical
Maximum displacement of the tibia (mm)	0.58	0.577	0.798	0.578	0.576	0.789
Gap deformation (mm)	0.224	0.219	0.445	0.222	0.216	0.442
Peak stress of the plate (MPa)	179.17	180.63	160.88	167	168.26	163.92
Peak stress of the screw (MPa)	135.81	137.51	530.88	137.29	139.78	541
Highest stress of the lateral hinge (maximum principle stress)	58.82	58.78	73.2	64.25	66.95	66.58
Lowest stress of the lateral hinge (maximum principle stress)	− 28.05	− 26.79	− 29.84	− 22.53	− 29.4	− 31.39

**Fig. 4:**
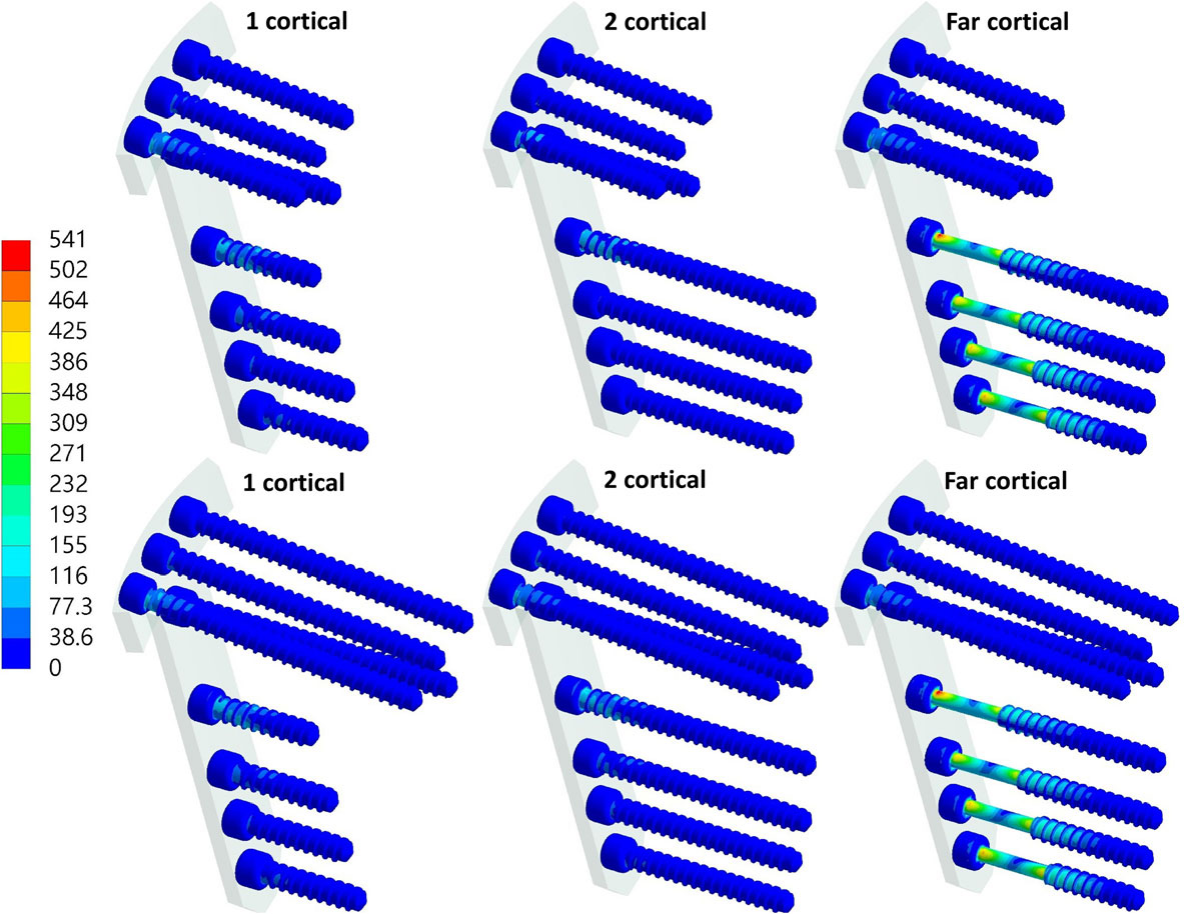
Equivalent stress of the screws with short (top row) and long (bottom row) screws at the proximal tibia

**Fig. 5:**
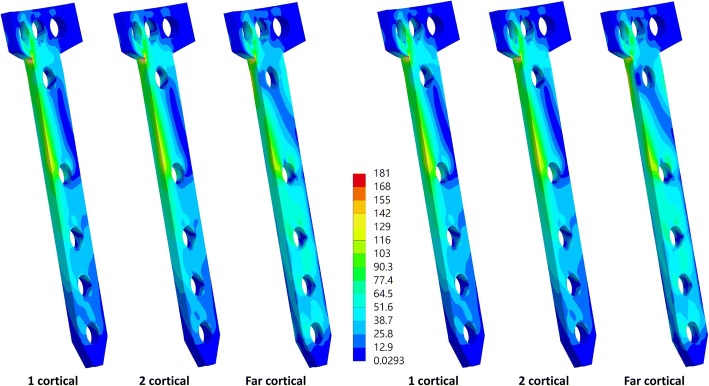
Equivalent stress of the plate with short (left) and long (right) screws at the proximal tibia

**Fig. 6:**
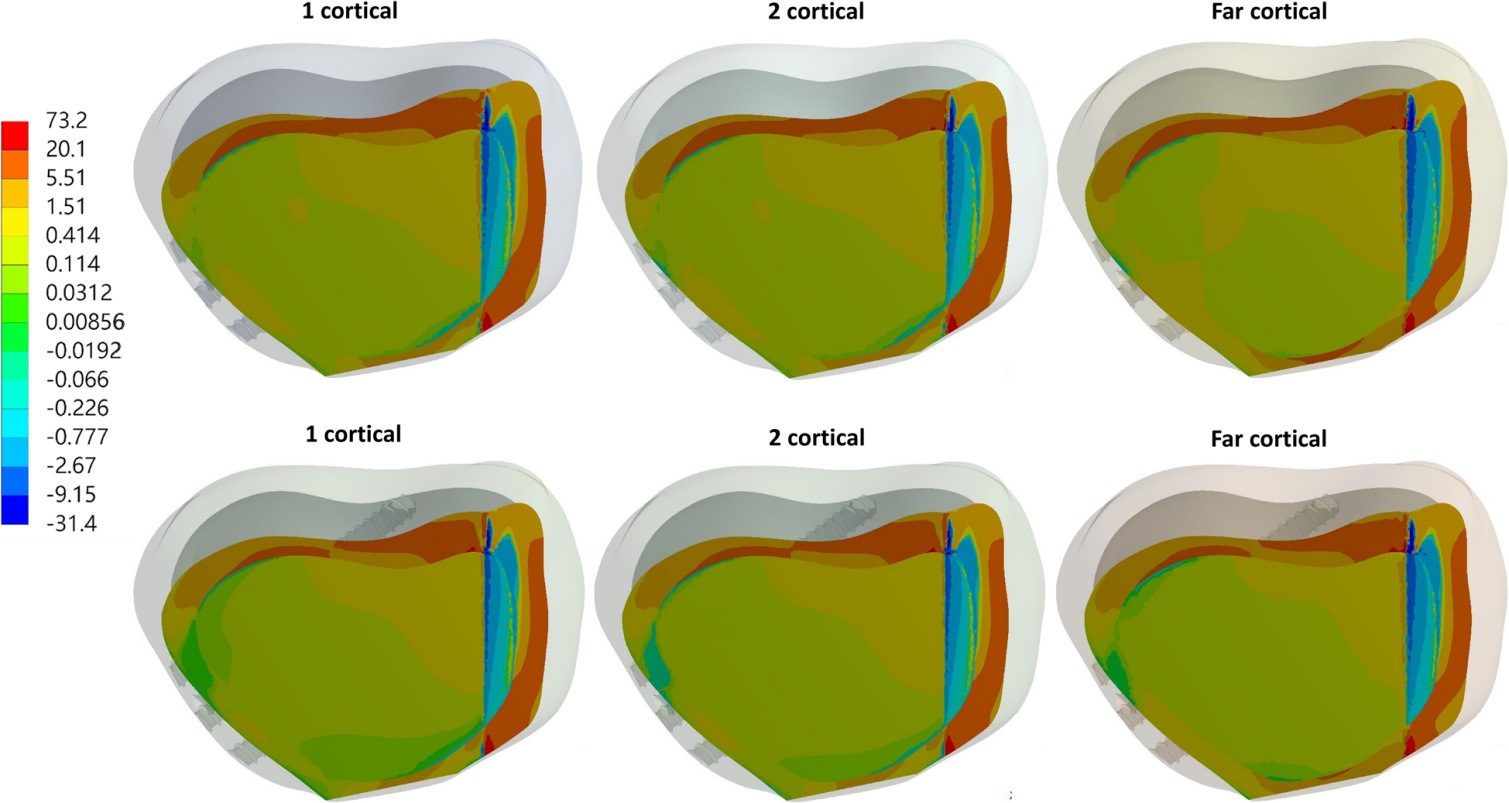
Maximum principle stress of the lateral hinge with short (top row) and long (bottom row) screws at the proximal tibia

The equivalent stress of the far-cortical locking screws was higher than that of the conventional locking screws (Fig. 4), with both one- and two-cortical locking, whereas the equivalent stress of the plate was slightly lower with the use of far-cortical locking screw than with the use of traditional locking screws (Table 2 and Fig. 5). The maximum equivalent stress of the far-cortical locking screw was 530.88 and 541 MPa with short and long screws, respectively, at the proximal tibia. At the proximal tibia, the far-cortical locking screw also resulted in higher maximum principle stress of the lateral hinge of the osteotomized tibia than one- and two-cortical locking screws (Table 2 and Fig. 6). The maximum equivalent stress of the lateral hinge with far- zcortical, two-cortical, and one-cortical screw was 73.2, 58.78, and 58.82 MPa, respectively.

## Discussion

This study investigated the mechanical responses of metallic implants (namely, the plate and screws) of various lengths, types, and configurations in HTO. The stress differences in the plate and screws depending on screw configurations were demonstrated. Furthermore, this study indicates the risk factor for screw failure in HTO with a titanium locking plate and various locking screws. The results can serve as a reference for surgeons performing HTO. A balance between stability and flexible fixation is essential.

The far-cortical locking screws and plate can decrease the stiffness of locking plates and screws and increase interfragmentary movements at the fracture or osteotomy site [31]. Furthermore, they promote callus formation during the bone healing process because of force stimulation at the fracture site. The decreasing diameter of the proximal shaft of the screw creates a gap between the screw and the cortex near the plate just after implantation without bone ingrowth (i.e., the screw hole is larger than the screw), which provides increased flexibility [13]. The FE simulations in this study and Roderer et al.’s study [11] have indicated that gap deformation is larger at the site when using HTO with flexible fixation than the conventional locking plate. Therefore, this technique is regarded as a potential solution for patients with delayed bone healing or nonunion after HTO.

The flexible fixation plate and screw increase interfragmentary movement, but the high screw stress demonstrated in the present FE model must not be ignored. The maximum equivalent stress of the screw in the far-cortical locking screw and plate system is higher than that in the conventional locking screw system. Because the proximal screw shaft of the far-cortical locking screw is thinner than that of the conventional locking screw and the proximal screw shaft does not contact with the surrounding bone because of a gap 0.75 mm, the thin screw must sustain the complete load. By contrast, the conventional locking screw is in contact with the tibia at the anterior region, and hence, the load is shared with the bones. Therefore, the equivalent stress of the far-cortical locking screw is higher than that of the conventional locking screw. The peak equivalent stress of the far-cortical locking screw (541 MPa) is less than the yield stress of titanium (800 MPa), but not substantially so. Owing to the high stress of the far-cortical locking screw, the risk of a far-cortical locking screw breakage is higher than that of a conventional locking screw after cyclic loadings. Moreover, the equivalent stress of the traditional locking screw is much lower than that of the far-cortical locking screw. Therefore, the risk of screw breakage is lower for conventional locking screws.

In clinical practice, the breakage of the lateral hinge during HTO is a matter of concern, and it may be multifactorial [32, 33]. In the present study, the maximum principle stress at the lateral hinge demonstrated no obvious difference between the different screw lengths and configurations. Further, based on the slightly increased maximum principle stress at the lateral hinge (approximately 24%, from 58.78 to 73.2 MPa), the authors believe the use of far-cortical locking screw does not play a major role in the breakage of the lateral hinge.

In Roderer et al.’s study, a dynamic locking screw system (DePuy Synthes, DePuy Synthes, Oberdorf, Switzerland) was used to increase the movement possible with the conventional locking screw at the osteotomy site. However, far-cortical locking screws and plates (MotionLoc Screw, Zimmer, Warsaw, USA) were used in this study because the dynamic locking screw system is not available in our country, and far-cortical locking screws are widespread. Furthermore, the intended use of dynamic locking screws and far-cortical locking screws is the same. Hence, far-cortical locking screw was used to replace the dynamic locking screw in this study.

We acknowledge some limitations in this study. First, the stress of the bone developed from the distracting process of the open wedge is not considered. Second, the thread at the proximal shaft of the far-cortical screw is neglected. Because the thread is for screw removal and does contact with the surrounding bone after fixation, smoothening the screw shaft does not affect the stability of the tibia in HTO. Third, only the maximum load in full knee extension was simulated, but the other knee positions and the muscle force were not considered. Fourth, all materials were simplified as isotropic and homogeneous.

## Conclusions

The study results suggest that far-cortical locking screws and plates can increase interfragmentary movement in HTO; however, the screw stress is relatively high. Increasing the protection time (partial weight duration) is suggested to decrease the risk of screw breakage in HTO through fixation with titanium far-cortical locking screws and plates.

## References

[1] Amis AA (2013). Biomechanics of high tibial osteotomy. Knee Surg Sports Traumatol Arthrosc.

[2] Lee DC, Byun SJ (2012). High tibial osteotomy. Knee Surg Relat Res.

[3] Losina E, Katz JN (2012). Total knee arthroplasty on the rise in younger patients: are we sure that past performance will guarantee future success?. Arthritis Rheum.

[4] Khan M, Evaniew N, Bedi A, Ayeni OR, Bhandari M (2014). Arthroscopic surgery for degenerative tears of the meniscus: a systematic review and meta-analysis. CMAJ.

[5] Akizuki S, Shibakawa A, Takizawa T, Yamazaki I, Horiuchi H (2008). The long-term outcome of high tibial osteotomy: a ten- to 20-year follow-up. J Bone Joint Surg Br.

[6] Ekhtiari S, Haldane CE, de Sa D, Simunovic N, Musahl V, Ayeni OR (2016). Return to work and sport following high tibial osteotomy: a systematic review. J Bone Joint Surg Am.

[7] Ha JK, Yeom CH, Jang HS, Song HE, Lee SJ, Kim KH, Chung KS, Bhat MG, Kim JG (2016). Biomechanical analysis of a novel wedge locking plate in a porcine tibial model. Clin Orthop Surg.

[8] Han SB, Bae JH, Lee SJ, Jung TG, Kim KH, Kwon JH, Nha KW (2014). Biomechanical properties of a new anatomical locking metal block plate for opening wedge high tibial osteotomy: uniplane osteotomy. Knee Surg Relat Res.

[9] Maas S, Diffo Kaze A, Dueck K, Pape D (2013). Static and dynamic differences in fixation stability between a spacer plate and a small stature plate fixator used for high tibial osteotomies: a biomechanical bone composite study. ISRN Orthop.

[10] Nelissen EM, van Langelaan EJ, Nelissen RG (2010). Stability of medial opening wedge high tibial osteotomy: a failure analysis. Int Orthop.

[11] Roderer G, Gebhard F, Duerselen L, Ignatius A, Claes L (2014). Delayed bone healing following high tibial osteotomy related to increased implant stiffness in locked plating. Injury.

[12] Bottlang M, Doornink J, Fitzpatrick DC, Madey SM (2009). Far cortical locking can reduce stiffness of locked plating constructs while retaining construct strength. J Bone Joint Surg Am.

[13] Doornink J, Fitzpatrick DC, Madey SM, Bottlang M (2011). Far cortical locking enables flexible fixation with periarticular locking plates. J Orthop Trauma.

[14] Stoffel K, Stachowiak G, Kuster M (2004). Open wedge high tibial osteotomy: biomechanical investigation of the modified Arthrex osteotomy plate (Puddu plate) and the TomoFix plate. Clin Biomech (Bristol, Avon).

[15] Luo CA, Hwa SY, Lin SC, Chen CM, Tseng CS (2015). Placement-induced effects on high tibial osteotomized construct - biomechanical tests and finite-element analyses. BMC Musculoskelet Disord.

[16] Luo CA, Lin SC, Hwa SY, Chen CM, Tseng CS (2015). Biomechanical effects of plate area and locking screw on medial open tibial osteotomy. Comput Methods Biomech Biomed Engin.

[17] Chen YN, Chang CW, Lin CW, Wang CW, Peng YT, Chang CH, Li CT (2017). Numerical investigation of fracture impaction in proximal humeral fracture fixation with locking plate and intramedullary nail. Int Orthop.

[18] Chen YN, Lee PY, Chang CH, Chang CW, Ho YH, Li CT, Peng YT (2016). Computational comparison of tibial diaphyseal fractures fixed with various degrees of prebending of titanium elastic nails and with and without end caps. Injury.

[19] Chen YN, Lee PY, Chang CW, Ho YH, Peng YT, Chang CH, Li CT (2017). Biomechanical investigation of titanium elastic nail prebending for treating diaphyseal long bone fractures. Australas Phys Eng Sci Med.

[20] Chang CW, Chen YN, Li CT, Chung YH, Chang CH, Peng YT (2018). Role of screw proximity in the fixation of transverse patellar fractures with screws and a wire. J Orthop Surg (Hong Kong).

[21] Vrancken AC, Crijns SP, Ploegmakers MJ, O'Kane C, van Tienen TG, Janssen D, Buma P, Verdonschot N (2014). 3D geometry analysis of the medial meniscus – a statistical shape modeling approach. J Anat.

[22] Weng PW, Chen CH, Luo CA, Sun JS, Tsuang YH, Cheng CK, Lin SC (2017). The effects of tibia profile, distraction angle, and knee load on wedge instability and hinge fracture: a finite element study. Med Eng Phys.

[23] Yang JC, Lin KP, Wei HW, Chen WC, Chiang CC, Chang MC, Tsai CL, Lin KJ (2018). Importance of a moderate plate-to-bone distance for the functioning of the far cortical locking system. Med Eng Phys.

[24] Butler DL, Kay MD, Stouffer DC (1986). Comparison of material properties in fascicle-bone units from human patellar tendon and knee ligaments. J Biomech.

[25] Goldstein SA, Wilson DL, Sonstegard DA, Matthews LS (1983). The mechanical properties of human tibial trabecular bone as a function of metaphyseal location. J Biomech.

[26] Townsend PR, Rose RM, Radin EL (1975). Buckling studies of single human trabeculae. J Biomech.

[27] Shirazi-Adl A, Dammak M, Paiement G (1993). Experimental determination of friction characteristics at the trabecular bone/porous-coated metal interface in cementless implants. J Biomed Mater Res.

[28] D'Lima DD, Patil S, Steklov N, Colwell CW (2007). An ABJS best paper: dynamic intraoperative ligament balancing for total knee arthroplasty. Clin Orthop Relat Res.

[29] Pauchard Y, Ivanov TG, McErlain DD, Milner JS, Giffin JR, Birmingham TB, Holdsworth DW. Assessing the local mechanical environment in medial opening wedge high tibial osteotomy using finite element analysis. J Biomech Eng. 2015;137(3):031005-1-7. 10.1115/1.4028966 25363041

[30] Kumar D, Manal KT, Rudolph KS (2013). Knee joint loading during gait in healthy controls and individuals with knee osteoarthritis. Osteoarthr Cartil.

[31] Heyland M, Duda GN, Haas NP, Trepczynski A, Dobele S, Hontzsch D, Schaser KD, Mardian S (2015). Semi-rigid screws provide an auxiliary option to plate working length to control interfragmentary movement in locking plate fixation at the distal femur. Injury.

[32] Nakamura R, Komatsu N, Fujita K, Kuroda K, Takahashi M, Omi R, Katsuki Y, Tsuchiya H (2017). Appropriate hinge position for prevention of unstable lateral hinge fracture in open wedge high tibial osteotomy. Bone Joint J.

[33] Lee OS, Lee YS (2018). Diagnostic value of computed tomography and risk factors for lateral hinge fracture in the open wedge high tibial osteotomy. Arthroscopy.

